# Supplementary material for: Highly Efficient Vertical Transmission for Zika Virus in Aedes aegypti after Long Extrinsic Incubation Time
Source: Pathogens. 2020 May 11;9(5):366. doi: 10.3390/pathogens9050366 (PMC7281418; doi:10.3390/pathogens9050366)
Supplement: Supplementary file 1 [file pathogens-09-00366-s001.pdf]

Supplementary Materials: The following are available online at www.mdpi.com/xxx/s1, Figure S1: Standard curve for absolute quantification of ZIKV gRNA. Table S1. Number of mosquitoes analyzed in the VT assay presented in Figure 3.

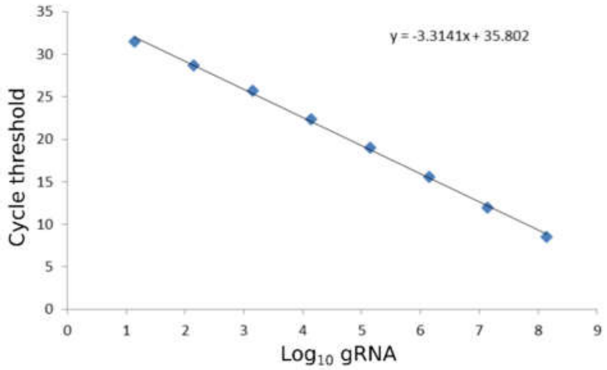

Figure S1. Standard curve for absolute quantification of ZIKV gRNA. Concentration of in vitro-transcribed RNA qPCR target was calculated and used to estimate gRNA copies. Ten-fold dilutions were used to calculate the equation between cycle threshold (Ct) and gRNA copies. The equation was then used to calculate gRNA copies in samples.

Table S1. Number of mosquitoes analyzed in the vertical transmission (VT) assay presented in Figure 3.

Condition	Rep.	Selected females	Infectious blood feeding	Oviposition at 3 d.p.i.	Blood feeding at 7 d.p.i.	Oviposition at 10 d.p.i.	Blood feeding at 14 d.p.i.	Oviposition at 17 d.p.i.
A	1	98	73	62	45	28	17	8
	2	147	99	72	36	25	14	9
B	1	71	Na	Na	Na	24	22	71
	2	100	Na	Na	Na	24	16	100

d.p.i.—days post oral infection; Na–Not applicable.
